# Map as a Service: A Framework for Visualising and Maximising Information Return from Multi-Modal Wireless Sensor Networks

**DOI:** 10.3390/s150922970

**Published:** 2015-09-11

**Authors:** Mohammad Hammoudeh, Robert Newman, Christopher Dennett, Sarah Mount, Omar Aldabbas

**Affiliations:** 1 School of Computing, Mathematics & Digital Technology, Manchester Metropolitan University, Manchester, M1 5GD, UK; 2 School of Mathematics and Computer Science, University of Wolverhampton, Wolverhampton WV1 1LY, UK; E-Mails: R.Newman@wlv.ac.uk (R.N.); C.Dennett@wlv.ac.uk (C.D.); s.mount@wlv.ac.uk (S.M.); 3 Faculty of Engineering, Al-Balqa Applied University, Salt, 12011, Jordan; E-Mail: o.aldabbas@bau.edu.jo

**Keywords:** Wireless Sensor Networks, information fusion, information extraction, information visualisation, service-oriented networks, mapping services, domain-model

## Abstract

This paper presents a distributed information extraction and visualisation service, called the *mapping service*, for maximising information return from large-scale wireless sensor networks. Such a service would greatly simplify the production of higher-level, information-rich, representations suitable for informing other network services and the delivery of field information visualisations. The mapping service utilises a blend of inductive and deductive models to map sense data accurately using externally available knowledge. It utilises the special characteristics of the application domain to render visualisations in a map format that are a precise reflection of the concrete reality. This service is suitable for visualising an arbitrary number of sense modalities. It is capable of visualising from multiple independent types of the sense data to overcome the limitations of generating visualisations from a single type of sense modality. Furthermore, the mapping service responds dynamically to changes in the environmental conditions, which may affect the visualisation performance by continuously updating the application domain model in a distributed manner. Finally, a distributed self-adaptation function is proposed with the goal of saving more power and generating more accurate data visualisation. We conduct comprehensive experimentation to evaluate the performance of our mapping service and show that it achieves low communication overhead, produces maps of high fidelity, and further minimises the mapping predictive error dynamically through integrating the application domain model in the mapping service.

## 1. Introduction

Wireless Sensor Networks (WSNs) offer the potential to give timely information in unattended environments. Dynamic visualisation of WSN data is needed to allow users to discover spatio-temporal patterns and intrinsic trends in such data. Information discovery in the ever-increasing collection of sensor data is an important problem in several scientific domains.

Developing a service that facilitates the extraction and representation of sense data presents many challenges. With sensors reporting readings at a very high frequency, resulting in large volumes of data, there is a need for a network service to efficiently extract and visualise sense data. Although considerable developments have been made towards low-cost extraction and visualisation of sensory data, these solutions remain inadequate. Most of the solutions available in literature address a specific data extraction or visualisation problem but ignore others (see Section 2.2). None of these solutions is able to simultaneously ensure low energy and memory consumption, while providing dynamic and real-time data extraction and visualisations. Thus, there is a need for a data extraction and visualisation framework that can balance the conflicting requirements of simplicity, expressiveness, timeliness, and accuracy with efficient resource utilisation. This paper presents an information extraction and visualisation service, called the mapping service, which solves global network data extraction and visualisation problems through localised and distributed computation.

Map as a Service (MaaS) offers great functionality such as the ability to analyse and geocode sensor data within spatial context. A service-oriented approach has special properties. It is made up of components and interconnections that stress interoperability and transparency. Services and service-oriented approaches address designing and building systems using heterogeneous network software components. This allows the development of the mapping service that works with any existing network components, e.g., routing protocols, and resources without adding extra overhead on the network.

Our investigation into the development of an efficient mapping service starts by describing a centralised implementation. The merits and shortcomings of the centralised mapping services are discussed. Then, challenges that need to be met before a distributed service is feasible are identified. The distributed mapping service views the network as an entity rather than a collection of separated nodes and uses only the existing data and network capabilities of a classical WSN. Such service is suitable for informing other network services as well as the delivery of field information visualisation. The final mapping framework extends the capabilities of the distributed mapping service to accommodate the particular characteristics of the application domain to improve the mapping performance. This is done by using inductive and deductive methods as well as the universal physical principles to map sense data. The mapping framework is suitable for mapping an arbitrary number of sensed modalities and is capable of mapping one sensed modality from related multiple types of sensed data to overcome the limitations of generating a map from a single type of sense modality.

This paper proceeds as follows. In Section 2, several mapping specific applications in the literature are outlined and a number of map generation techniques for WSNs are reviewed. Section 3 describes the mapping service for data extraction and visualisation in WSNs. A centralised implementation of this service is presented and its merits and shortcomings are discussed. Section 4 is the main exposition of the distributed map generation algorithm. It explains how to exploit other network services to provide more efficient, yet, less expensive mapping solutions. Section 5 presents the integrated inductive-detective framework for sensor data mapping. Section 6, presents the experimental evaluation of the proposed mapping service. Finally, conclusions are drawn and further work is suggested.

## 2. Related Work

### 2.1. Mapping Applications in the Literature

Within the WSN field, mapping applications found in the literature are ultimately concerned with the problem of mapping measurements onto a model of the environment. Estrin [1] proposed the construction of isobar maps in WSNs and showed how in-network merging of isobars could help reduce the amount of communication. Furthermore, Meng *et al.* [2] proposed an efficient data-collection scheme, and the building of contour maps, for event monitoring and network-wide diagnosis, in centralised networks. Solutions, such as distributed mapping, have been proposed to the general mapping domain. However, many solutions are limited to particular applications and constrained with unreliable assumptions. The grid alignment of sensors in [1], for example, is one such assumption.

In the wider literature, mapping was sought as a useful tool in respect to network diagnosis and monitoring [2], power management [3], and jammed-area detections [4]. For instance, contour maps were found to be an effective solution to the pattern matching problem that works for limited resource networks [5]. Rather than resolving these types of isolated concerns, in this work the WSN is expected not only to produce map type responses to queries but also to make use of the data supporting the maps for more effective routing, further intelligent data aggregation and information extraction, power scheduling and other network processes.

These are examples of specific instances of the mapping problem and, as such, motivate the development of a generic distributed mapping framework, furthering the area of research by moving beyond the limitations of the centralised approaches.

### 2.2. Algorithms for Map Generation

Map generation techniques have previously been explored in the context of WSNs [5,6]. Chang *et al.* [6] implemented an algorithm to estimate sensor nodes faulty behaviour on top of a cluster-based network. This approach is based on Bayesian Belief Networks, which make it problematic to compute all the probabilities and the revised probabilities once a new sensor reading is received. In high density networks and when nodes can sense more than one modality, the number of dependencies increases rapidly and probabilities computation becomes an NP-hard problem.

Event detection based on matching the contour maps of in-network data distribution has been shown effective for event detection in WSNs [5]. This technique was based on the observation that events in WSNs can be abstracted into spatio-temporal patterns of sensory data and pattern matching can be done efficiently through contour map matching. Using these contour maps as building blocks, events based on the spatio-temporal patterns exhibited in the contour maps are defined. This technique works well with grid network topologies and less well with random topologies, which may be more common in real life applications. When a grid is overlaid on top of a random topology, some cells in the grid may be empty. This makes the scheme sensitive and unsuitable for random sensor networks deployments.

Matching contour maps from sensor observations is not the only method of event detection in WSNs, although it is the closest approach to the one proposed by this paper. A number of methods have been devised, which use sensor data directly. For example, [7] uses the number of events detected by individual sensors as a test statistic to determine the overall probability that an event has occurred. However, this technique is computationally expensive. Probabilistic techniques such as [7,8,9] are promising, but have been verified by simulation, and make a number of assumptions about the sensing behaviour of nodes (such as the ideal sensors rule in [9]) and the communication stacks. At present, it is difficult to predict how such methods will perform in practice, particularly in the presence of energy restrictions.

The distributed mapping service, proposed in Section 6, has been partly inspired by the work of [10]. Their work made the case for a large-scale distributed multi-resolution storage system that provides a unified view of data handling in WSNs incorporating long-term storage, multi-resolution data access and spatio-temporal correlations in sensor data. This work is related to ours, but different in focus at both the system architecture and coding level. It outlines an approach for relatively power-rich devices, focused on encoding regularly-gridded, spatial wavelets over time series. In contrast, we focus on resource constrained devices. Our work also focuses on spatially deployed networks and is independent on a particular routing or interpolation algorithm.

In centralised map generation approaches, delivering all network sensory data back to the sink incurs heavy transmission traffic, which quickly depletes the energy of sensor nodes and causes bandwidth bottlenecks. Several aggregation based map generation methods have been proposed to address this problem [2,5,11,12]. However, aggregation based methods can not further improve the scalability of the network as all sensors are required to report to the sink. Moreover, the aggregation process increases the computation overhead on the intermediate nodes, as studied in detail by [13,14].

Our proposed map generation solution is similar in spirit to the one presented in [2]. The work in [2] (for short we refer to it as suppression-based mapping) utilises suppression techniques to reduce the number of nodes reporting their raw readings to the sink. It trades accuracy with the amount of samples. The scheme consists of three algorithms to build contour maps: distributed spatial and temporal data suppression, centralised contour reconstruction via interpolation and smoothing, and a mechanism for multiple hop routing. As in our approach, the interpolation algorithm utilises knowledge of the terrain of the sensor field and the location of the sensors to accurately reconstruct the contour maps. This scheme is based on the dumb sensor, smart sink paradigm, which imposes little processing and storage overhead on nodes. Therefore, it is not suitable for the delivery of field information visualisations. However, in this paper we are interested in in-network data processing to produce low cost maps. Nevertheless, our algorithm targets networks with limited capabilities, i.e., sensors have limited storage space and computational power. In the lack of recent applicable approaches to compare the performance of our work against, we choose the suppression-based mapping.

To address the inherent limitations of aggregation based methods, Liu and Li [15] proposed a method called *Iso-Map* that intelligently selects a small portion of the nodes, *isoline nodes*, to generate and report mapping data. Partial utilisation of the network data leads to a decrease in the mapping fidelity and isoline nodes will suffer from heavy computation and communication load. Furthermore, the location of the mapping nodes can also affect the directions of traffic flow and thereby have a significant impact of the network lifetime. Finally, in sparsely deployed low density networks it is difficult to construct contour maps based only on isoline nodes. The positions of isoline nodes provide only discrete *iso-positions*, which does not define how to deduce how the isolines pass through these positions.

To conclude, mapping is often employed in WSN applications, but as yet there is no clear definition (or published work towards) a distributed mapping framework that would aid the development of more sophisticated services. The development and analysis of such a framework is the key novel contribution of the work presented here.

## 3. A Centralised Mapping Service

In essence, map generation is a problem of interpolation from discrete and irregular points. Given a set of known data points representing the nodes’ perception of a given measurable parameter of the phenomenon, what is the most likely complete and continuous map of that parameter? In the field of computer graphics, this problem is known as an disorganised points problem, or a cloud of points problem. That is, since the position of the points in xy is assumed to be known, the third parameter can be thought of as height and surface reconstruction algorithms can be applied. Simple algorithms use the point cloud as vertices in the reconstructed surface. These are not difficult to calculate, but can be inefficient if the point cloud is not evenly distributed, or is dense in areas of little geometric variation.

Approximation, or iterative fitting algorithms define a new surface that is iteratively shaped to fit the point cloud. Although approximation algorithms can be more complex, the positions of vertices are not bound to the positions of points from the cloud. For applications in WSNs, this means that we can define a mesh density different to the number of sensor nodes, and produce a mesh that makes more efficient use of the vertices. Self organising maps are one of the algorithms that can be used for surface reconstruction [16]. This method uses a fixed number of vertices that move towards the known data.

Note that surface reconstruction on typical non-overlapping terrains is equivalent to sparse-data interpolation. This kind of geometric parameter interpolation has been shown to work well for reconstructing underlying geography when the entire network has been queried [17]. However, it does not extend well to variable surfaces or overlapping local mapping, since it requires a complete data set to define the surface. A more general method is interpolation by inverse distance and, specifically, Shepard Interpolation [18] which improves on it. This algorithm has been implemented and used by the mapping service to interpolate between sensor readings.

There has been a consistent effort to change the mechanism of use of certain capabilities in WSN, to simplify and abstract them, turning them into services of the network rather than being the result of coordinating the services of individual nodes. Although, the primary purpose of WSN is to collect and transmit data, but other capabilities have arisen to support this goal. Just as clustering, routing and aggregation allow for more sophisticated and efficient use of the network resources, the mapping service would support other network services and make many more applications possible with little extra effort. Following on from such work and moving to a slightly higher level of abstraction, we propose a new network service: *Map Generation*. This Map Generation service is studied in detail in Section 3.1.

In this simple centralised mapping service, all data is transmitted to the sink and a global map is computed at once. This approach will produce high quality maps as the entire network data is used for building the map. However, a fully centralised data collection is not always feasible. Data needs to travel to a central point, which possibly causes bandwidth bottlenecks especially around the sink. This also may cause large drains on resources. Furthermore, a big portion of this return data is not useful, particularly the unchanged modalities and redundant packets. Also, this method usually does not scale with the network size and requires high communication overhead to maintain up-to-date maps. Finally, in some scenarios, it is desirable or necessary to process this information on site and, as a result, in-network mapping provides a critical solution to in-field data analysis.

The centralised mapping service is a composition of three core network components: the Map Generation service, the routing service, and the mapping applications. The Map Generation service encapsulates the new network capability, interpolation. The Map Generation makes it easy to predict values at points where there is no sensing device. The routing service encapsulates the communication architecture used for data delivery to the sink. The mapping service can be on top of any routing protocol, however, in this work, the ROL/NDC routing protocol [19] was used for data communication. ROL/NDC has proven to be an energy efficient and robust routing protocol. It is already implemented, which allowed the authors to direct the implementation efforts on the Map Generation service. Finally, the application module contains the entire code specific to the user application functionality. Section 6.1 presents a sample mapping application.

### 3.1. A New Network Service: Map Generation

Often, WSNs field data collection takes the form of single points that need to be processed to get a continuous map. The problem of map generation is essentially a problem of interpolation from sparse and irregular points. Interpolation describes the process of taking many single points and building a complete surface, the inter-node gaps being filled based on the spatial statistics of the observation points.

Through extensive empirical analysis and research, published in [20], we identified the Shepard interpolation algorithm [18] as a suitable algorithm for the distributed mapping service. Visual aspects, sensitivity to parameters, and timing requirements were used to test the characteristics of this method. Shepard interpolation was found to be an attractive solution due to its wide use in the literature. It is intuitively understandable and provides a large variety of possible customisations to suit particular purposes, e.g. relaxation of the interpolation condition for noisy data sets [21]. Also, Shepard’s method can be easily modified to incorporate different external conditions that might have an impact on the interpolation results, such as physical barriers. Shepard’s method is simple to implement with fast computation and modelling time [22,23] and generalisation to more than two independent variables is feasible. This method can be localised, which is an advantage for large and frequently changing data sets, making it suitable for sensor network applications. Local map generation by Shepard function reduces data communication across the network and evades the computation of the complete network map when one or more observations are changed. Finally, there are few parameter decisions and it makes only one assumption, which gives it the advantage over other interpolation methods [22].

Shepard defined a continuous function where the weighted average of data is inversely proportional to the distance from the interpolated location. The algorithm explicitly implies that the further away a point is from an interpolated location, *P*, the less effect it will have on the interpolated value. This algorithm exploits the intuitive sense that things that are close to each other are more alike than those that are further apart.

Data points, $D_{i}$, are weighted during interpolation relative to their distance from *P*. Weighting is assigned to data through the use of a weighting power, which controls how the weighting factors drop off as the distance from *P* increases. Shepard’s expression for globally modelling a surface is:

(1) $$f_{1}\left(P\right)=\left\{\begin{array}{cc} \frac{\sum_{i=1}^{N}\left[{\left(d_{i}\right)}^{-u}z_{i}\right]}{\sum_{i=1}^{N}{\left(d_{i}\right)}^{-u}} & \mathrm{if}d_{i}\neq 0\;\forall \;D_{i}\left(u>0\right)\\ z_{i} & \mathrm{if}d_{i}=0\mathrm{for}\mathrm{some}D_{i} \end{array}\right.$$

where $d_{i}$ is the standard distance metric from *P* to the point *D* numbered *i* in the *N* known points set and $z_{i}$ is the known value at point $D_{i}$. The exponent *u* is used to control the smoothness of the interpolation. As the distance between *P* and $D_{i}$ increases, the weight of that sampled point will decrease exponentially. As *P* approaches $D_{i}$, $d_{i}$ tends to zero and the *i*th terms in both the numerator and denominator exceeds all bounds while other terms remain bounded. Therefore, the $lim_{P\to D_{i}}f_{1}\left(P\right)=z_{i}$ is as desired and the function $f_{1}\left(P\right)$ is continuously differentiable even at the junctions of local functions.

### 3.2. Discussion

The centralised approach collects all of the data in a central location and processes it there. In networks of a few nodes, this might even be the most sensible solution. However, in networks of nodes large enough to be useful, the number of transmissions needed to accomplish the task would become so large as to require a more intelligent solution. The cost of communication in this initial system is high because the interpolation step; the Mapping Generation service is fairly simple and requires that all data is collected to a point. For the simplest case, in which the node collecting data is in direct communication with all other nodes in the network, the number of hops is simply N-1, for *N* nodes. However, this is an unlikely situation in any real world application. The likelihood is exponential growth with *N*.

Although the cost, in terms of power of transmission of data in WSNs far outweighs the usual processing load, the nodes are most often power-limited devices with, at best, a fixed schedule of battery replacement, and so computational drain on this resource can not be completely overlooked. For small collections of data of around 50 nodes interpolating 65,536 points—a square field (256×256)—takes very little time, just a few seconds, on a desktop computer and would take just a little more on modern sensor nodes. In a real deployment, the number of sensor nodes might easily exceed 1000, depending upon the required density, drastically increasing computational expense. The acceptability of such a computational load in a WSN is debatable, but in this case would probably be unacceptable for most deployments.

The simplest improvement is to develop a complete implementation of Shepard’s refinements and assess how this affects the accuracy of the interpolated field when compared to a known real surface or phenomenon. Although the algorithm presented by Shepard is decades old, it is often only implemented in its most simple form and it would be of great benefit to have empirical evidence of the performance of the various improvements suggested by Shepard, when applied to data likely to be perceived by a sensor network. A particularly interesting feature of the refinements is that they deal with limiting the data included in the weighted average (the support set), or adjusting weights based on context. These improvements might be instantly applicable as ways to reduce communication costs as well as improve the quality of the interpolation. That is, if local information is required, then only local nodes need be queried. Taking this idea further, we arrive at the idea of a distributed mapping service that allows for local querying.

## 4. A Distributed Mapping Service

The distributed construction of accurate and efficient visualisations of sense data is an open problem. This section presents a solution in which groups of network nodes cooperate to produce local maps that are cached and merged at the sink node, producing a low-resolution map of the global network. The sink node receives periodic map updates from each cluster head used to refine an up-to-date global map. The global map gives a low cost interface that can be used to target queries for generating detailed maps from a subset of sensors in the network. In an energy constrained network, it is preferable to allow the application to control the level of detail and topology coverage of a generated map.

Solving global network data mapping problems through localised and distributed computation is achieved by abstracting and simplifying the routing and the Map Generation services and turning them into services of the network rather than being the result of coordinating the services of individual nodes. This scheme changes the mechanism of use of certain capabilities in WSNs; the Map Generation service is used by sensor nodes to deal with continuous representation of sense data and to localise map computation; while cluster heads are selected as the caches for local maps.

**Figure 1:**
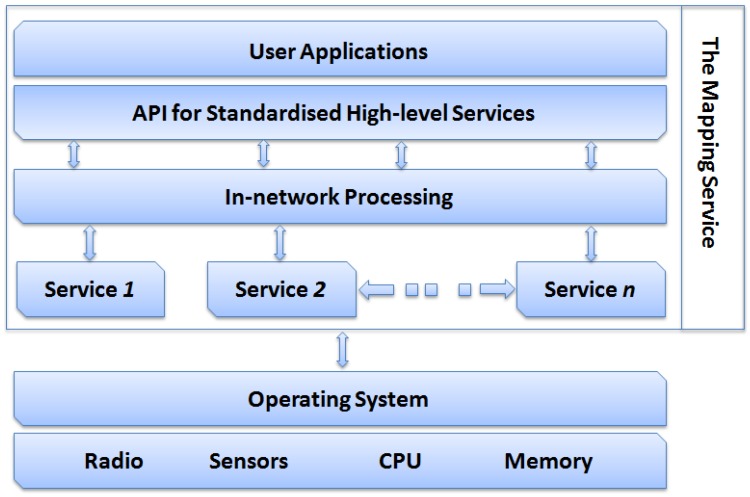
The architecture of the distributed mapping service.

Figure 1 shows the distributed mapping service architecture and interactions between its component. It is made up of four layers:

1. The *Network Services Layer*: This layer hosts the services of the network. In this work, the mapping service exploits the Map Generation and the routing services. However, this layer may contain other services such as, power management and self-healing.
2. The *In-network Processing* layer: The role of this layer is to process raw data received from various cluster heads. It applies filtering on all the received data to reduce redundancy resulting from overlapping cluster coverage. Moreover, the *In-network Processing* module manages incremental update messages and merges them into a single transaction. Also, it defines interfaces for the *Network Services Layer* and the *API for Standardised High-level Services* layers. It also provides access to the cached data in a suitable format.
3. The *API for Standardised High-level Services*: This layer provides the essential components and interfaces that facilitate the development and deployment of end-user mapping applications, e.g. isopleth or terrain navigation applications. This is a simple layer that does not include the more complex features and capabilities required for building a middleware.
4. The *User Applications* layer: This layer hosts the user mapping applications.

### 4.1. An Algorithm for Distributed Map Construction

The major steps for the distributed construction of the multi-resolution map are as follows.

#### 4.1.1. Cluster Head Collects Data From Its Vicinity

Sensed data is sent to the cluster head in the form of a tuple (x,v,t) where *x* is the 2D Cartesian coordinates of the sensing node, *v* is the value of the measured parameter(s), and *t* is a time-stamp denoting the time at which the data packet was created. If a sensor node is in the initial data collection phase, it computes a local accuracy model, T, and buffers all its sensors readings. In latter data transmission phases, a sensor node classifies each new reading according to the local accuracy model and then sends its cluster head only significant readings. T can be simply a pre-defined threshold that when exceeded it signifies an important change in the state of the monitored condition or it can be a more complex model.

Though attempting to achieve a desired global objective, sensor nodes interact with each other only within a restricted neighbourhood, defined using the Map Generation service. A sensing node only buffers data packets for neighbouring nodes that have a significant effect on its local map. For instance, Shepard’s method defines an arbitrary number of nodes, *N*, within a particular radius, *R*, from the node as the set of nodes which has effect on the local map. For more information about how *N* and *R* are calculated see [18].

To avoid sending large amounts of data across the network, various cluster heads collect data from their members and create summary data sets as defined by the In-network Processing model. The size of a summary data set is determined according to the accuracy level required by the complete network map. These summary messages are then transmitted to the sink.

#### 4.1.2. Cluster Head Computes Its Partial Map and Forwards It to the Sink

The cluster head merges received data and compress it by removing redundant points. The data compression process makes the interpolation step faster. Given an up-to-date data set, the cluster head can easily generate a partial map. The cluster head caches two data representations: point data and graphical map. The former is used to build the local graphical map that is forwarded to the sink to be combined with data received from other clusters to build the complete network map. While the latter is used to respond quickly to user queries about information within the cluster area.

#### 4.1.3. The Sink Computes Complete Network Map

The sink fuses the partial map received from all cluster heads into a complete map of the network. By applying the same interpolation step performed at the local cluster heads, the sink generates a complete map of the network. When requested by the user, this map can be refined by querying a more detailed map from the cluster head(s) managing the area of interest.

#### 4.1.4. Dynamically Update the Partial and Complete Maps

In many real-world applications of WSNs, the sensed modalities are mostly unchanged over time [5]. One method for updating maps in the presence of such changes is for each node to use the Map Generation service to build a local accuracy model, which can be used by the node to base its decisions of reporting a change in its local map to its cluster head. The local accuracy model is defined such that a node is able to estimate its own reading using other observation points within some level of tolerance. This level of tolerance can be derived from the global cluster map accuracy. When the accuracy level changes sufficiently, the sensor node saves the new calculated map using the most recent readings and it reports these changes to its corresponding cluster head. If several updates arrive at the cluster head about the same time, they are grouped into one update transaction before being sent to the sink. Algorithm 1 outlines this approach.
**Algorithm 1** Incremental update of local and global maps.**IF** node is a sensing node **THEN** create a local accuracy model classify each new reading according to the local accuracy model **IF** local accuracy deviates **THEN**  update the local map  forward the new reading to its cluster head **END IF****END IF****IF** node is a cluster head **THEN** **WHEN** receive update message  update local map  send the update message to the sink**END IF**sink combines up-to-date local maps


**Figure 2:**
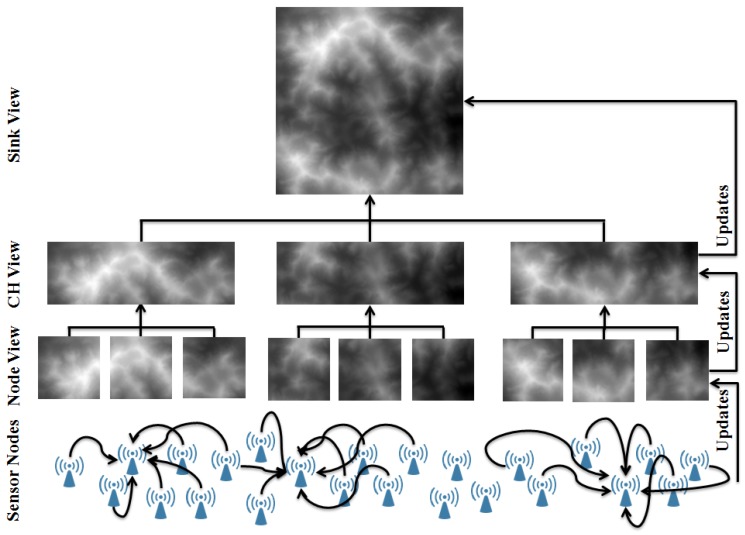
An illustrative diagram showing a generic WSN and how the distributed mapping service operates on it.

Figure 2 shows how the proposed approach operates on a generic WSN. Sensor nodes at the bottom layer of the network collect data from their neighbours to build their support set, which is fed into the mapping services to generate the nodes local view. Then, sensor nodes forward their mapping data to their cluster head, where it is merged to generate the cluster view. Maps from all clusters are merged at the sink to generate the global map. Maps at different levels are updated progressively from bottom all the way up to the sink.

## 5. An Integrated Inductive-Deductive Framework for Data Mapping

The real worth of the enormous amount of data generated by the WSN lies not only in easy data access, but also in the additional possibility of extracting as well as presenting the implicit information contained in the data. Recently, such networks are being deployed for an increasingly diverse set of applications each with different characteristics and environmental constraints. As a consequence, scientists from different research fields have begun to realise the importance of identifying and understanding the characteristics and special deployment needs of different application domains. In many WSN deployments, the network owners have some knowledge about the monitored environment characteristics in which the target system operates. For example, in forest fire applications, information about the forest topography can be obtained from the Geographic Information System (GIS) or satellites maps.

WSNs have an intimate interaction, via sensors, with the physical environment they operate within because they are usually deployed to perform application specific tasks. The part of the world with which an application is concerned is defined as that application’s domain. This work advocates that an application domain of a WSN can serve as a supplement to data analysis, interpretation, and visualisation methods and tools. Therefore, we elevate the distributed mapping service capabilities to make use of the special characteristics of applications. This section extends the distributed mapping service to an Adaptive, Multi-modal Application domain driven (AMA) mapping framework, which is suitable for mapping an arbitrary number of sense modalities and is capable of utilising the relations between different modalities as well as other parameters of the application domain to improve the mapping performance. AMA is capable of dealing with dynamically changing application domain conditions. It starts with an initial user defined model that is autonomously maintained and updated throughout the network lifetime.

A domain model carries knowledge of an application domain. It is a conceptual model of a system, which describes the various real world entities involved in that system and relationships between them. The domain model provides a structural view of the system, which we suggest using to complement the information gained from analysing data gathered by a WSN. In this section, we argue that by using additional knowledge available about the monitored environment, the distributed mapping service has a greater potential for giving more meaning to the collected data. The logical integration of a domain model and sensory data from multiple heterogeneous sensory sources can be effectively used to explain past observations as well as to predict future observations. This approach can take advantage of human guidance and information from other available sources, e.g., satellites. Furthermore, it maintains the overall coherence of reasoning about the gathered data and helps to estimate the degree of confidence using probabilistic domain models. The use of knowledge made available by the domain model can also be key to meeting the energy and channel capacity constraints of a WSN system. The energy efficiency of the system can be improved by utilising a domain model in the process of converting data into increasingly distilled and high-level representations.

### 5.1. AMA Framework Details

**Figure 3:**
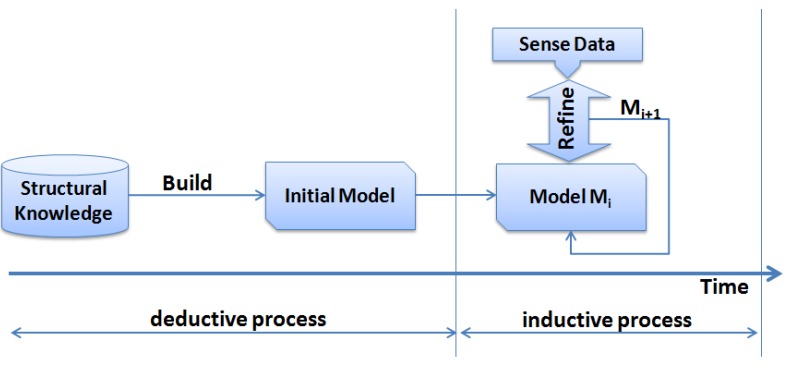
The process of building the domain model by deductive and inductive steps.

AMA utilises a blend of both inductive and deductive models to establish a successful mapping between the sensed data and the universal physical principles. For example, in WSNs forest fire application, factors such as the wind speed and direction, topography, and the type of forest have a big influence on the application. Some of these factors can not be sensed or the deployed hardware platform does not have suitable sensing equipment to gather this information. However, this information is very easy to acquire from external resources such as the GIS, geology databases, and weather stations, and can be integrated into the application to assure the consistency of the theory with the experimental data collected by the network. Therefore, it is desirable to combine the advantages of both methods.Deductive methods rely on a precise application environment and the explicit knowledge, called structural knowledge, of the underlying domain using first principles to create a model of the problem typically yielding governing equations [24]. On the other hand, inductive methods utilise experimental data as the only source of available knowledge [24]. Some applications can only be treated using experimental data knowledge due to the lack of other application domain knowledge. Nevertheless, the use of inductive information helps in the generation of data consistency checks based on the structural knowledge abstractions given in the domain model. However, applications have been observed to perform significantly better if they use a combination of the two methods [25]. Figure 3 shows how the inductive and deductive methods can be merged to capture the advantages of both methods. After the structural knowledge is fed into the system model (deductive process), the sensed data is used to refine and complement the basic structural model of the application domain. This refinement procedure can be done continuously throughout the network life to keep consistent mapping between the physical model and the sensed data.

AMA performs mapping from related multiple types of sense data to overcome the limitations of generating a map from a single sense modality. A single sense modality map typically reveals only a small number of aspects of the monitored phenomena and is unable to infer the correct relations among other types in multi-modal sensing applications. In addition to this inherent data deficiency, high-throughput data sets also often contain errors and noise arising from imperfections of the sensing devices, which further obstructs mapping effectiveness. Maps generated from a combination of different types of data are likely to lead to a more coherent map by consolidating information on various aspects of the monitored phenomena. Additionally, the effects of data noise on generated maps will be dramatically reduced, assuming that sensing errors across different data sets are largely independent and the probability that an error is supported by more than one type of data is small. A natural approach to making use of the relation between the multiple types of sensed data to generate a map is to combine the maps generated from different types of data. We may combine the maps in different methods such as accepting a value at an observation point only when it is commensurate with all maps as defined in the given model. More interestingly, and perhaps with guarantees of delivering better maps, multiple types of data can be analysed concurrently under an integrated relational model. The latter method is novel in the sense that most existing *n*-dimensional interpolation schemes are defined by applying one-dimensional interpolation in each separate coordinate dimension without taking advantage of the known relations between diverse dimensions [26].

#### 5.1.1. Multi-Modal Spatial Interpolation

Most spatial data interpolation methods are based on the distance between *P*, where the interpolation function has to be determined, and the given set of data points. The AMA defines a new metric for distance, suitable for higher dimensions, in which the concept of closeness is described in terms of relationships between sets rather than in terms of the Euclidean distance between points. Using this distance metric, a new generalised interpolation function *f*, that is suitable for an arbitrary number of variables, is defined.

In multi-dimensional interpolation, every set $S_{i}$ corresponds to an input variable, *i.e.*, a sense modality, called *i*, and referred to as a dimension. In AMA, the distance function does not need to satisfy the formal mathematical requirements for the Euclidean distance definition. The power of such a generalisation can be seen when we include the time variable as one dimension. The spatial data interpolation problem can be stated as follows:

Given a set of randomly distributed data points

(2) $$x_{i}\in \Omega ,\;i\in \left[1,N\right],\;\Omega \subset R^{n}$$

with function values $y_{i}\in R$, and $i\in \left[1,N\right]$, we require a continuous function f:Ω⟶R to interpolate unknown intermediate points such that

(3) $$f\left(x_{i}\right)=y_{i}\;\mathrm{where}\;i\in \left[1,N\right]$$

We refer to $x_{i}$ as the observation points. The integer *n* is the number of dimensions and Ω is a suitable domain containing the observation points. When rewriting this definition in terms of relationships between sets we get the following:

Given *N* ordered pairs of *separated* sets $S_{i}\subset \Omega$ with continuous functions

(4) $$f_{i}:S_{i}⟶R,\;i\in \left[1,n\right]$$

we require a multi-dimensional continuous function f:Ω⟶R, defined in the domain $\Omega =S_{1}\cup S_{2}\cup ...\cup S_{n-1}\cup S_{n}$ of the *n*-dimensional Euclidean space where

(5) $$f\left(x_{i}\right)=f_{i}\left(x_{i}\right)\forall x_{i}\in S_{i}\;\mathrm{where}\;i\in \left[1,n\right]$$

The existence of the global continuous function *f* can be verified as follows. First, the data set is defined as

(6) $$S=\left\{\begin{array}{cccc} v_{1}^{\left(0\right)}, & v_{1}^{\left(1\right)}, & \cdots , & v_{1}^{\left(n^{\prime }\right)}\\ v_{2}^{\left(0\right)}, & v_{2}^{\left(1\right)}, & \cdots , & v_{2}^{\left(n^{\prime }\right)}\\ \vdots & \vdots & & \vdots \\ v_{n}^{\left(0\right)}, & v_{n}^{\left(1\right)}, & \cdots , & v_{n}^{\left(n^{\prime }\right)} \end{array}\right\}$$

where $n^{\prime }\leq N$ and $v_{i}=\left(x_{i},r_{i}\right),i\in \left[1,N\right]$ and $r_{i}$ is a reading value of some distinctive modality (e.g., temperature). Let Φ be a topological space on *S* and there exists open subsets $S_{i},i\in \left[1,n\right]$

(7) $$\begin{array}{c} S_{1}=\left\{v_{1}^{\left(0\right)},v_{1}^{\left(1\right)},\cdots ,v_{1}^{\left(n^{\prime }\right)}\right\}\\ S_{2}=\left\{v_{2}^{\left(0\right)},v_{2}^{\left(1\right)},\cdots ,v_{2}^{\left(n^{\prime }\right)}\right\}\\ \vdots \\ S_{n}=\left\{v_{n}^{\left(0\right)},v_{n}^{\left(1\right)},\cdots ,v_{n}^{\left(n^{\prime }\right)}\right\} \end{array}$$

which are topological subspaces of Φ such that

(8) $$\Phi_{S_{i}}=\left\{S_{i}\cap U|U\in \Phi \right\}$$

Also define Ψ as a topological space on the co-domain R of function *f*. Then, there exists a function, *f*, that has the following properties:

1. Let $f:S_{1}\cup S_{2}\cup ...\cup S_{n-1}\cup S_{n}$ be a mapping defined on the union of subsets $S_{i},i\in \left[1,N\right]$ such that the restriction mappings $f_{|S_{i}}$ are continuous. If subsets $S_{i}$ are *open* subspaces of *S* or *weakly* separated, then there exist a function *f* that is continuous over *S* (proved by Karno [27]).
2. If f:Ω→Ψ is continuous, then the restriction to $S_{i},i\in \left[1,N\right]$ is continuous (property, see [28]). The restriction of a continuous global mapping function to a smaller local set, $S_{i}$, is still continuous. The local set follows since open sets in the subspace topology are formed from open sets in the topology of the whole space.

Using the point to set distance generalisation, the function *f* can be determined as a natural generalisation of methods developed for approximating uni-dimension function. Well-known uni-dimension interpolation formulas can be extended to the multi-dimension case by using *Geometric Algebra* (GA) in a special way while using a point to set distance metric. Burley *et al.* [29] discuss the usefulness of GA for adapting uni-dimension numerical methods to multi-dimension data using no additional mathematical derivation. Their work was motivated by the fact that it is possible to define GAs over an arbitrary number of geometric dimensions and that it is therefore theoretically possible to work with any number of dimensions. This is done simply by replacing the algebra of the real numbers by that of the GA. We apply the ideas in [29] to find a multivariate analogue of uni-variate interpolation functions. To show how this approach works, an example of Shepard interpolation of this form is given below:

Given a set of *n* distinct points $X=\left\{x_{0},x_{1},...,x_{n}\right\}\subset R^{s}$, the classical Shepard’s interpolation function is defined by

(9) $$\left(S_{n,\mu }^{o}f\right)\left(x\right)=\sum_{k=0}^{n}w_{k}\left(x\right)f\left(x_{k}\right)$$

and

(10) $$w_{k}\left(x\right)=\frac{|x-x_{k}{|}^{-\mu }}{\sum_{k=0}^{n}{\left|x-x_{k}\right|}^{-\mu }}$$

where |.| denotes the Euclidean norm in $R^{s}$.

In the uni-variate case $\left(s=1\right)\;and\;S_{n,2}^{0}f$. The basic properties of $S_{n,\mu }^{0}f$ are:

1. $\left(S_{n,\mu }^{0}f\right)\left(x_{i}\right)=f\left(x_{i}\right),i=0,...,n;$
2. $\mathrm{doe}\left(S_{n,\mu }^{0}f\right)=0$, where doe is an abbreviation of degree of exactness.

#### 5.1.2. Scale-Based Local Distance Metric

In this section, we modify the distance metric defined in Shepard interpolation to include the knowledge given by the domain model. The domain model helps to significantly reduce the size of the support nodes set to lower than that of the conventional Euclidean-distance-based interpolation methods. The increase in the size of the support set can lead to an increase in the computation and processing times of the interpolation algorithm. Therefore, the proposed metric attempts to balance the size of the support set with the interpolation algorithm complexity as well as interpolation accuracy.

We define the term *scale* for determining the weight of every given dimension with respect to *P* based on a combined Euclidean distance criteria as well as information already known about the application domain a priori to network deployment. While the term *weight* is reserved for the relevance of a data site by calculating the Euclidean distance between **P** and a particular data point. A special case is when $f_{i}$ is identical for all $S_{i}$ which means that all sets have the same scale.

We define a new scale-based weighting metric, $m_{P}$, which uses the information given by the domain model to alter the distance weighting function to improve the interpolation results when applied to an arbitrary number of dimensions. All data sets are ordered pairs $\left(x_{i},y_{i}\right)$ such that $x_{i}$ is the sampling location and $y_{i}$ is a measured scalar value associated with $x_{i}$.

Given a set of data points $S_{i}$, standard spatial data interpolation methods define the interpolation function based on the distance from *P* to $C_{i}$ where $C_{i}\subseteq S_{i}$. In local interpolators, $C_{i}$ is a small collection of the total set of data points. When $C_{i}$ is extended to include a large proportion of the data points or the total set of points the interpolation function becomes global. In AMA, the set $C_{i}$ for each dimension is determined using $m_{P}$. Symbolically, $C_{i}$ is calculated as

(11) $$\begin{array}{cc} C_{i}=L\left(d\left(P,E_{j}\right),\delta \left(S_{i}\right)\right) & \forall E_{j}\in S_{i} \end{array}$$

where $i\in \left[1,n\right]$, *L* is a local model that selects the support set for calculating *P*, *d* is an Euclidean distance function, $E_{j}$ is an observation point in the dimension $S_{i}$, and $\delta (S_{i})$ a set of parameters for dimension $S_{i}$. Each dimension can have a different set of parameters. These parameters are usually a set of relationships between different dimensions or other application domain characteristics such as obstacles. When predicting the value of a point in dimension $S_{i}$ we refer to that dimension as $S_{P}$.

In uni-dimensional distance weighting methods, the weight, ω can be calculated as follows

(12) $$\omega =d\left(P,E_{j}\right),E_{j}\in S_{i}$$

This function can be extended to multi-dimensional distance weighting systems as follows

(13) $$\begin{array}{cc} \omega =K\left(P,S_{i}\right), & i\in [0,n] \end{array}$$

where $K\left(P,S_{i}\right)$ is the distance from *P* to data set $S_{i}$ and *n* is the number of dimensions in the system. Equation (13) can now be extended to include the domain model parameters of arbitrary dimensional system. Then, the dimension-based scaling metric can be defined as

(14) $$\begin{array}{cc} m_{P}=\sum_{i}L\left(K\left(P,S_{i}\right),\delta \left(S_{i}\right)\right) & i\in \left[0,n\right]\;and\;S_{i}\neq C_{P} \end{array}$$

where $C_{P}$ is the dimension containing *P*.

### 5.2. Distributed Self-Adaptation in AMA

In this subsection, we extend the capabilities of AMA to overcome challenges imposed by external system changes through applying self-adaptation intelligence to continuously adapt to erratic changes in the application domain conditions. Dynamically adaptive mapping includes tasks that detect external system changes and tasks that update the domain model.

We realise that self-adaptation is a challenging problem and considerable work is being done by the research community in that area. However, in this work we aim to deal with a small set of adaptivity issues that have a significant effect on the mapping service.

#### 5.2.1. Benefits of Self-Adaptation

Self-adaptation is particularly useful in long-term WSN deployments, where the environmental conditions change significantly over time. Such changes necessitate the update of the domain model to reflect changes in the contextual knowledge provided by the physical constraints imposed by the local environmental conditions where sensors are located. Self-adapting mapping will provide high reliability and predictability for years at a time without constant tuning of the domain model by experts. This allows the mapping service to evolve at run-time with less intervention of the user and leads to near-optimal and flexible design that is simple and inexpensive to deploy and maintain. This adaptation procedure will recover, over time, from the effects of user domain modelling inaccuracies.

#### 5.2.2. Adaptability Implementation in AMA

To implement adaptability in AMA, the interpolation capability of the network is exploited to perform local training of the Map Generation service. Each node uses the readings of its surrounding nodes to predict its own reading value $\left(y^{\prime }\right)$ using the *scale-based metric* defined in Equation (14). Then, $y^{\prime }$ is compared to the node measured value (y). It is desirable that the estimate of $y^{\prime }$ minimises the Standard Deviation σ.

Nodes modify the size of the support set to include the minimum number of nodes needed to predict *y* with a certain level of accuracy. Furthermore, in multi-dimensional applications, nodes will change the weight of each dimension to improve the prediction accuracy of $y^{\prime }$. In fact, nodes will alter the relationships between different dimensions given in the initial domain model to reduce the effect of modelling inaccuracies or to adapt to emerging environmental changes. These model updates influence the estimation results, because $y^{\prime }$ is calculated using the scaling metric $m_{p}$ (Equation (14)). Finally, a prediction accuracy criterion, Δ, is defined as the average $\sigma_{j}$ where

(15) $$\sigma_{j}=\sum_{i}\sqrt{{\left(y_{i}-y_{i}^{\prime }\right)}^{2}}\;j\in \left[1,n\right]\;and\;i\in \left[1,N\right]$$

where *n* is the number of dimensions and *N* is the number of readings in dimension *j*. Then, Δ is written as

(16) $$\Delta =\frac{\sum \sigma_{j}}{n}\;j\in \left[1,n\right]$$

Likewise the one dimensional case, Δ must always be minimised to achieve the best mapping results.

However, when individual nodes alter their programmed domain model independently from the network, the Mapping Service may become unstable because of the inconsistency in the domain model defined on various nodes. Such inconsistencies may lead to inconsistent system states and conflicting differences in calculating mapping values. Furthermore, some of the detected environmental changes may result from sensing faults, which are hard to detect without collaboration with neighbouring nodes. To ensure mapping stability and overcome such concerns, we propose a *Virtual Congress Algorithm* to manage global model updates locally.

#### 5.2.3. The Virtual Congress Algorithm

The *Virtual Congress Algorithm* (VCA) top-down technique is proposed to manage the global and local domain model updates. VCA provides a high-level collaboration environment in which the system can achieve globally efficient behaviour under dynamic environmental conditions. Instead of re-programming individual nodes, the network is viewed as a virtual congress where nodes are *senators* who vote for legislating changes to the domain model in response to locally detected environmental conditions. This algorithm is an attractive solution as senators collaboratively decide upon their local knowledge on the behaviour and correctness of the system. Logically related nodes, *chambers*, are granted some power to impute the local changes, *federal decisions*, that is not detected by all nodes in the network. In cluster-based networks, a cluster can be defined as a chamber. A senator may introduce a proposal in the chamber as a *bill*. To prevent overloading the chamber with proposals, each senator must monitor the changes over time using Equation (16) before putting them into a bill. Besides, no two or more identical bills are allowed to be proposed by two different senators. Senators evaluate and study the proposed bill and send their voting results accordingly to the proposing senator. Upon receiving the required number of votes *υ*, the proposing senator disseminates the bill to the chamber and all nodes implement the new changes that have been agreed on. The value of *υ* was empirically estimated to be over 50% of chamber population because it helps to avoid false positives (see Experiment 6.3.3 for empirical analysis). If the bill gets rejected, then the proposing senator suppose that there is a local problem and starts a fault detection or recalibration service. Once a bill is approved by one chamber, it is sent to other chamber heads who may accept or reject it. In order for the bill to become a *state law*, all chamber heads must agree to identical version of the bill. When the bill is submitted to the *president*, the sink node, he may choose to sign the bill, thereby making it *law*. The president informs all chamber heads whether the bill became a law or not. If the chamber head does not receive a response from the president within a specific period of time, then it register the bill it generated as rejected.

**Figure 4:**
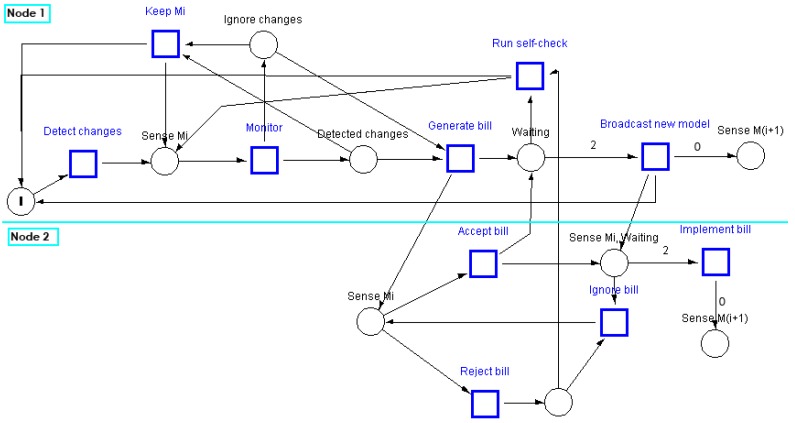
A Petri-net that models the VCA.

Figure 4 shows a Petri net that models the behaviour of the VCA in a chamber that consists of two sensing nodes (senators). In this model the first senator, Node 1, detects a change in the environmental conditions. The place with a single token is a guarding condition that allows the senator to generate only one bill at any time. When a senator detects a change, it enters into a monitoring phase after which it decides to keep the existing local domain model or to generate a bill to update that model. When a senator collects the required number of votes (in this scenario υ=2), it broadcasts the new model which is then implemented by all nodes in the chamber. Finally, the token is deposited into the start place after the completion of the model update activity.

This Petri net model was simulated using the S/T Petri-Net simulation system [30]. The simulation of the VCA Petri net with non-deterministic selection of activities allows checking the algorithm against deadlocks caused by controllers, introduced to enforce the given specifications, especially in the closed-loop net branches. It was found that the VCA Petri net is live and deadlock-free.

## 6. Experimental Evaluation

### 6.1. Experimental Isopleth Generation

Using contour lines (also isoline or isopleth) to represent the distribution of data is a common technique when there exists a trade-off between having more information about the data and affording higher costs to collect the additional information [2]. To highlight the potential use of the mapping service, an implementation of isopleth generation is presented. This is a simple implementation, with little regard for efficiency, but nevertheless provides a genuinely useful output for the purposes of visualisation or phenomenon edge delineation.

A user makes a request to any node, giving the value of the required isopleth. A threshold figure, *t*, is also given so that values ±t are included. The threshold *t* of the isopleth specifies the desired isolines in the isopleth map with the isolevels. The simple algorithm presented here causes this node to then query every other node in the network for their value of the parameter in question. Once all data is collected, the map generation is performed, and every value matching the required isopleth is recorded as being part of that isopleth. An isopleth for a given parameter at a given value can be found by a step-by-step search for the next point in the map at the same level. That is, a single point is used as the start of the isopleth and the next is found by searching the neighbourhood in progressively more detail. The process is repeated until the end of the isopleth moves out of the mapped region or meets the beginning. The highest level of detail that is searched will determine how fine the isopleth is.

**Figure 5:**
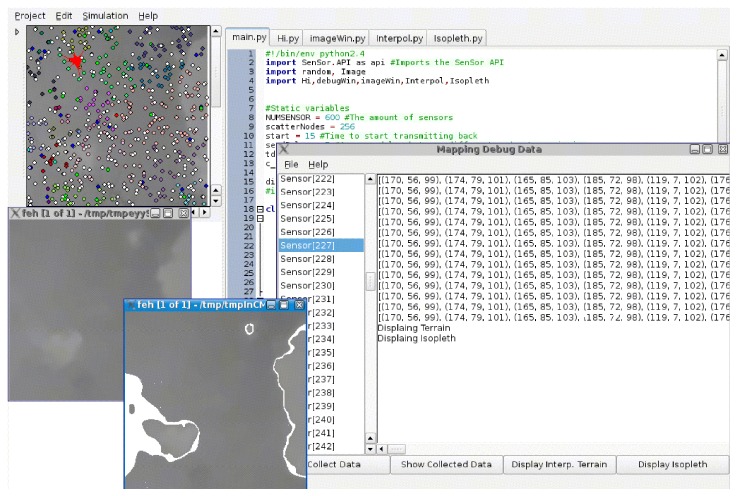
Dingo WSN simulator.

**Figure 6:**
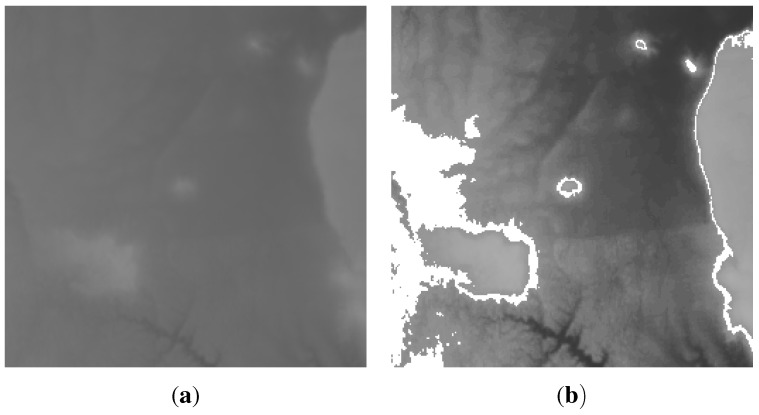
(**a**) A section of the Grand Canyon height map; (**b**) A contour at 116 units drawn over 2D map in (**a**).

**Figure 7:**
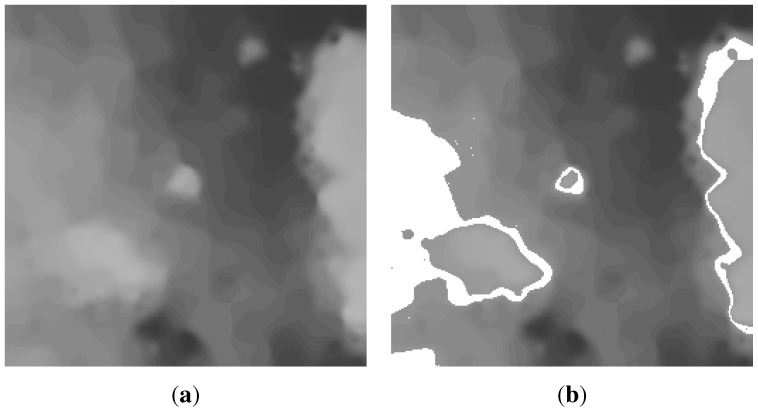
(**a**) Field generated from 600 nodes randomly distributed on the surface of the 2D map in Figure 6a; (**b**) A contour at 116 units drawn on the mapped field in (**a**).

To implement this algorithm, the Dingo [31] WSNs simulator was used. A section of the Grand Canyon height map [32], is fed into the simulator (see Figure 5). The height map was chosen because using numerous wide-distributed height points has been an important topic in the field of spatial information [33]. Furthermore, the height is a static measure, which makes it suitable for the evaluation of various mapping algorithms. The studied region is $15,360\;m^{2}$, with heights ranging from 165m to 284m above sea level. This map was sampled to 65,536 points. The sensor nodes were randomly distributed over the height map. The grey value of each pixel denotes the altitude of that point. If the isopleth generation algorithm is applied to this data directly, the result is shown in Figure 6. This contour was generated for a height of 116 units and a threshold of 1. Figure 7a shows the field generated by the centralised mapping service from 600 nodes randomly distributed on the surface of the 2D map in Figure 6a. The result of generating an isopleth on the interpolated field with the same parameters as in Figure 6b, are shown in Figure 7b.

Clearly the interpolated terrain is similar to the real surface. Determining exactly how close the similarity is, and what the algorithmic limits to the accuracy of the representation, is a problem we investigated in [20]. Consider, though, that the information used to reconstruct the surface in Figure 7 is just 600 points, while the original is recorded as 65,536 points. Taking the position of the nodes into account as extra information, the reconstruction is built using less than 1% of the original data. The cost of mapping using the centralised mapping services is plotted; it is used as base line scheme for comparison with the distributed mapping service.

The same improvements intended to decrease the cost of transmission also reduce the cost of computation. The change from global to local area mapping reduces the number of transmissions and makes the task of map generation significantly smaller. Additionally, since the local mapping could be performed by local nodes, the computational cost is not just reduced, but also distributed. That is, rather than individual nodes taking big losses in battery life and becoming unusable quickly, the entire network node’s batteries degrade at a more constant rate.

### 6.2. The Distributed Mapping Service

**Figure 8:**
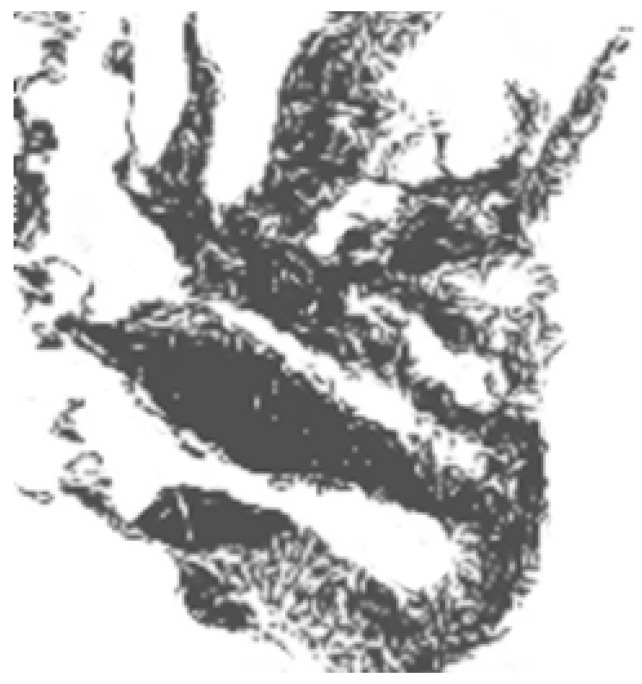
A mineral map derived from AVIRIS data.

In this experiment, the efficiency of the distributed mapping service in terms of energy consumption and the quality of the produced map is studied by simulating its execution on a mineral map, see Figure 8, derived from AVIRIS data obtained over Cuprite, Nevada, in 1995 [32]. The satellite spatial resolution is about 17 m pixel spacing and the scene is 4.5 km wide and 4.5 km high and north is up. In this experiment, 2000 nodes were randomly distributed over the area described above. The results obtained here are compared to the results of the centralised mapping service and the results of the suppression-based mapping. The latter approach is the most recent published work, which achieves the best fidelity compared with all other existing approaches. For both approaches, node density is the dominating factor affecting the accuracy of the contour mapping. Therefore, we simulate different node densities to reflect this impact.

**Figure 9:**
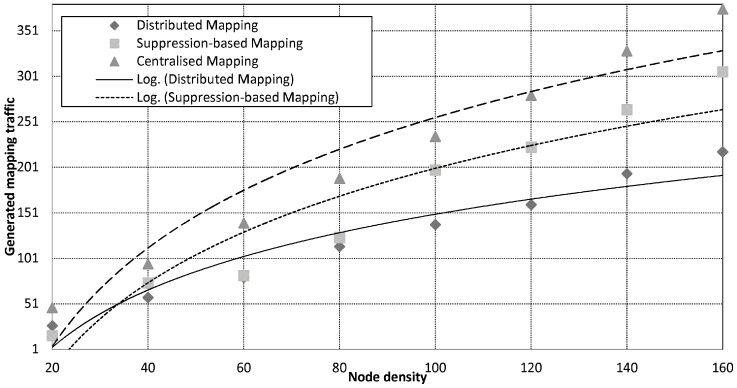
The cost of map generation against node density.

#### 6.2.1. Energy Consumption

Energy consumption is classified as follows: energy needed to distribute a query, energy needed to transmit readings, and energy needed to receive readings. Figure 9 plots mapping traffic against the number of nodes in the network. Overall, the distributed mapping service saves significant amount of energy compared to the centralised mapping service. Evidently, delivering all readings back to the sink incurs heavy traffic, which depletes the energy of sensor nodes. The traffic overhead incurred by centralised mapping grows rapidly and scales proportional to the number of nodes in the network, O(n). Suppression-based mapping reduces the traffic exchange through spatial and temporal suppression techniques. Although the utilisation of in-network suppression techniques implements mapping with reduced traffic cost, it does not reduce the scale of the generated traffic. This is because of the additional cost introduced by the broadcast of the hash functions created by the sink to every data sources to enable multi-hop suppression. When data exhibits low spatial correlation, the suppression technique becomes less effective resulting in higher traffic returned to the sink. Moreover, energy for overhearing neighbours turns out to be the dominant factor in the overall energy consumption. In the distributed scheme, the global map is built from locally computed partial maps without relying on communications with the centralised sink. To avoid excessive communication, partial maps are continuously updated locally avoiding complete re-computation of the global map. Unlike suppression-based mapping, the distributed mapping service scales fairly well with respect to the network size and achieves these energy saving without requiring any extra set-up messaging (e.g., hash functions). Based on data in Figure 9, the distributed mapping scheme reduces the mapping traffic by up to 70% and 30% compared to the centralised mapping and the suppression-based mapping schemes respectively.

#### 6.2.2. Quality of the Reconstructed Maps

**Figure 10:**
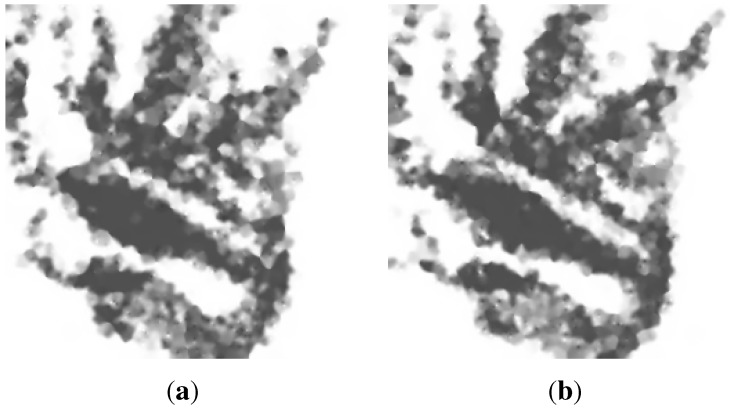
Maps generated from 2000 nodes randomly distributed on the surface from Figure 8 using the centralised and distributed mapping services respectively.

**Figure 11:**
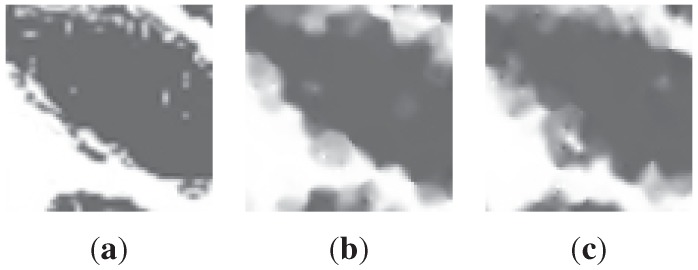
A section from Figure 8 and Figure 10a,b respectively.

Figure 10 show the maps constructed by the centralised and the distributed mapping services respectively. The two maps were generated assuming no packet loss. By comparing the two maps, it is obvious that they are visually very similar to each other as well as to the original map. This demonstrates that the distributed mapping service offers accuracy level comparable to that of the centralised service. Quantitatively, the difference between the high-resolution source data and the re-constructed data is measured as the root-mean-square error (RMSE) difference of the pixels. Using this measurement, there is just a 1.2% difference between the distributed and centralised mapping algorithms. This is because the two mapping services utilise the full network data, which naturally leads almost equal mapping fidelity. One particular side-effect of the distributed method can be beneficial. The enforced exclusion of distant points in local mapping can produce a final map that is visually much closer to the original for some features. The influence of irreverent distant points in the centralised mapping (Figure 11b) causes less of a similarity with Figure 11a than the distributed method (Figure 11c). Note the complete absence of the “loop” shapes under the main dark region in Figure 11b.

**Figure 12:**
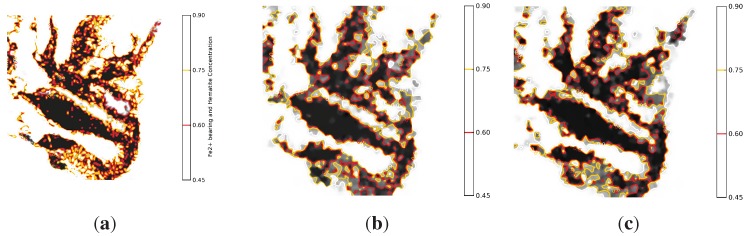
Contour maps generated by the three studied mapping schemes.

Figure 12 shows the isopleth maps reconstructed by using the three studied mapping schemes. Without imposing any measurement noise, all maps were constructed for a 116 units and a threshold of 1. The distributed and suppression-based contour maps deviate from the centralised contour map by different degrees. By comparing the two maps, the distributed mapping service outperforms the suppression-based mapping. This is because the used mineral map exhibits low spatial correlation. In all studied mapping schemes, the limited capability of the underlying wireless communications contributes to the degradation of the mapping fidelity.

### 6.3. AMA

While no single domain of scientific endeavour can serve as a basis for designing a general framework, an appropriate choice of specific application domain is important in providing insights relating to performance of the mapping service. Therefore, to illustrate the benefits of exploiting the domain model in map generation we consider heat diffusion in metals objects. Heat diffusion is important for a variety of real-life applications, e.g., cargo ship fires. This application domain contains many interesting layout and environmental features, such as hatches, which allows thorough testing of the AMA.

The studied domain model was restricted to a chamber in the ship that has one large hatch. The chamber was modelled by a brass sheet that contains a hole excavation to model an opened hatch (heat diffusion obstacle). Brass (an alloy of copper and zinc) was chosen because it is a good thermal conductor and it allows imaging within the temperature range of the available Infrared (IR) camera with less reflection than other metals such as Aluminium and Steel. A FLIR ThermaCAM P65 IR camera [34] was used to take highly accurate heat images. These images serve as a base-line to measure the accuracy of the generated maps.

#### 6.3.1. Experiment 1: Incorporation of the Domain Model in Mapping

This experiment aims to study the effect of integrating the domain model knowledge into the mapping service.

**Figure 13:**
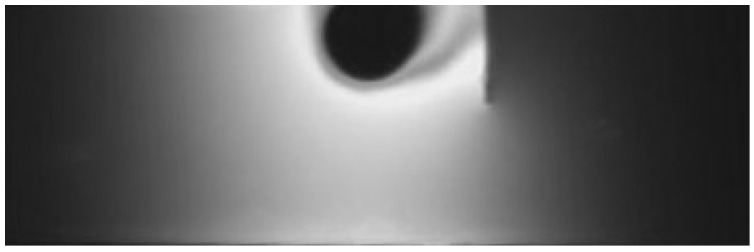
Heat diffusion map taken by the IR camera.

A heat source was placed on the sheet edge to the left of the obstacle. After 30 s of applying heat, a thermal image for the sheet was taken by the IR camera. Figure 13 shows this heat diffusion map. It can be observed that the obstacle strongly reduced the temperature rise in the area on the right side of the brass sheet. This map has been randomly down-sampled to 1000 points, that is 1.5% of the total 455×147 points. The sampled set is used by the AMA to re-generate the complete thermal map by three different mapping runs: (1) The mapping service does not have any domain model knowledge (behaves as the standard distributed mapping service); (2) The mapping service integrates *some* knowledge given by the domain model. Particularly, the presence of the obstacle, its position, and length; (3) The mapping service integrates *all* the knowledge given by the domain model (all information from run (2) plus the obstacle strength.)

**Figure 14:**
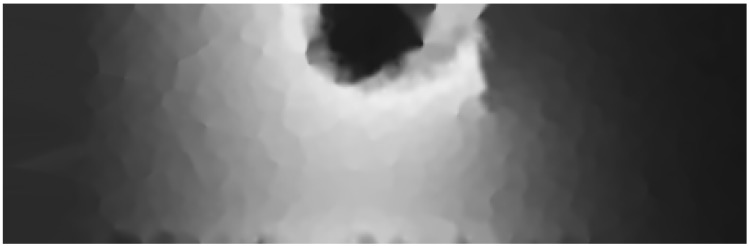
Heat map generated by run (1).

Figure 14 shows the map generated by run (1). Compared with Figure 13, the obtained map conserves perfectly the global appearance and many of the details of the original map with 98.5% less data. However, the area containing the obstacle has not been correctly reconstructed and hard edges appeared around the heat source. This is due to the physical discontinuities between adjacent sensors, which attenuates the logical relationship between them. The fact that some interpolation areas contain many sensor readings with almost the same elevation also contributes to distortions in the map quality.

**Figure 15:**
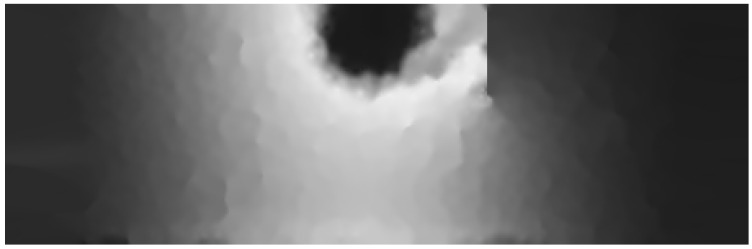
Heat map generated by run (2).

Figure 15 shows the map generated by run (2). In this run, AMA reduced the prediction error compared to run (1). Particularly, AMA accurately captured the effect of the obstacle. The AMA generated map is smoother than that rendered with the distributed mapping service, especially in some sub-regions containing the obstacle and around the heat source location. Moreover, AMA knowledge about the length of the obstacle reduced the heat diffusion prediction error in the area on the right of the obstacle, which was not captured accurately in run (1).

**Figure 16:**
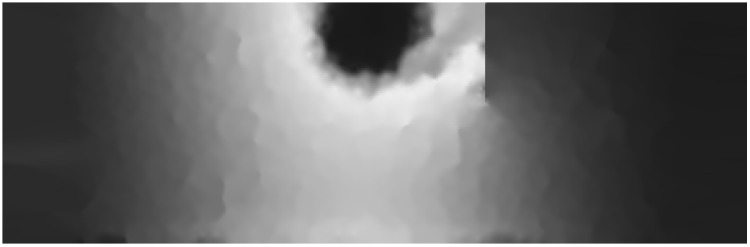
Heat map generated by run (3).

**Figure 17:**
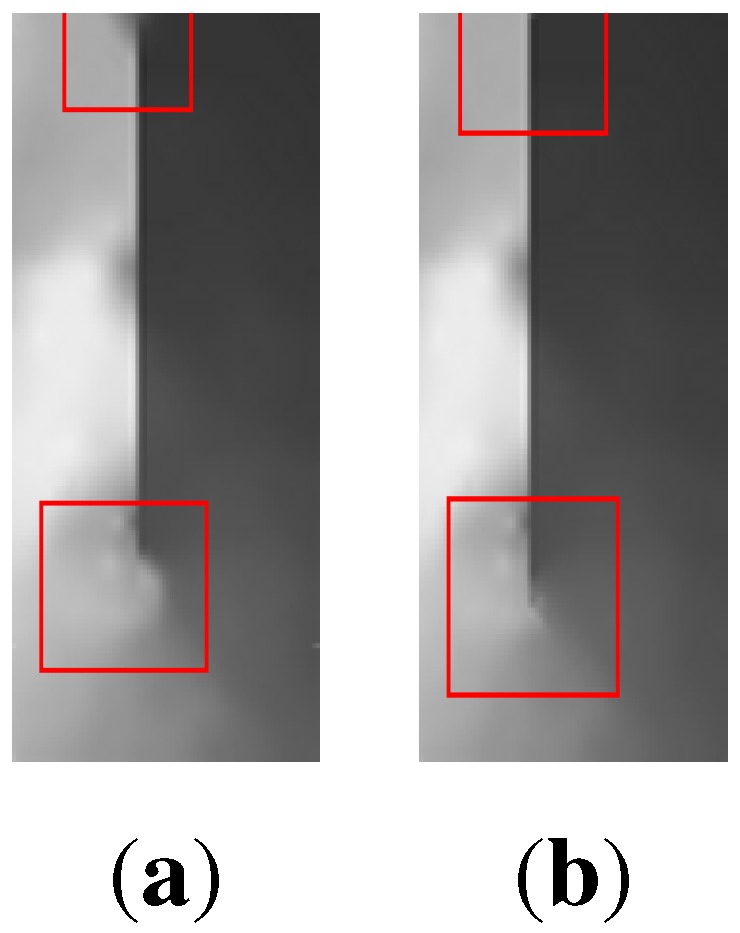
(**a**) Detailed section of Figure 15 showing the area around the obstacle with artefacts marked in red. (**b**) Detailed section of Figure 16 showing the area around the obstacle with reduced artefacts.

Figure 16 shows the map generated by run (3). A better approximation to the real surface near the obstacle is observed. The new details included in the domain model removed some artefacts from both ends of the obstacle as shown in Figure 17. This is due to the inclusion of the obstacle width in weighting sensor readings when calculating *P*, which further reduced the effect of geographically nearby sensors that are separated from *P* by the obstacle.

These experiments show that the incorporation of the domain model in the mapping service significantly improves the performance of the distributed mapping service. It achieves better mapping quality at a lower cost by further excluding nearby nodes that are logically unrelated to the interpolated location.

#### 6.3.2. Experiment 2: Mapping from Related Multiple Dimensions

The aim of this experiment is to study how mapping from related independent sensory modalities can lead to an improved mapping performance by overcoming some of the limitations of generating a map from a single sensory modality.

**Figure 18:**
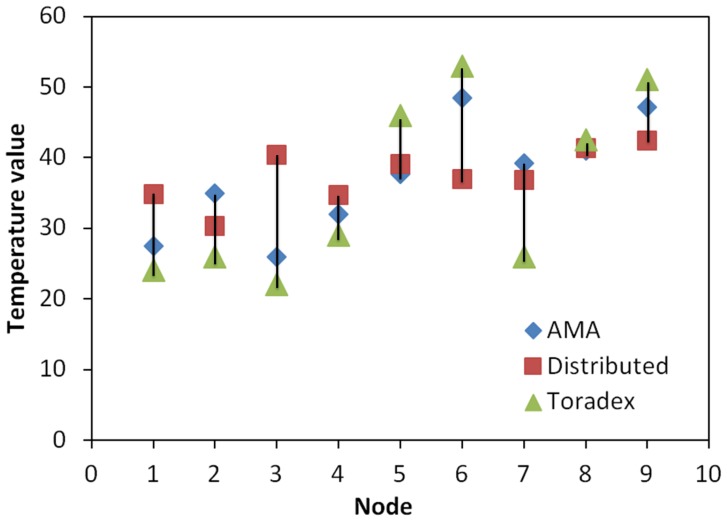
The temperature values calculated by the distributed mapping service and by AMA compared to the actual reading.

In this experiment, ten Toradex Oak USB Sensor [35] nodes equipped with humidity and temperature sensors were deployed on the brass sheet. Cold water was sprayed onto the brass sheet to increase the humidity in order to make the relationship between the temperature and humidity more visible. After 30 s of applying heat, thermal and humidity measurements from the sensor nodes were recorded. Then, for each node, the temperature reading was removed from the collected data set one at a time. Both the distributed mapping service and the AMA were used to calculate the removed temperature reading using the rest of the data set. Figure 18 plots the two calculated and the measured temperature readings.

With the presence of only one surface discontinuity, AMA reduced the percent error by 11.8% compared to the distributed mapping service. The percent error is the relative change calculated from the absolute change between the actual (measured) and determined (accepted) values divided by the accepted value [36].

AMA uses the scale-based local distance metric to further reduce the effect of unrelated and uncertain measurements. It weights independent temperature measurements and adjusts the support set by changing the relative importance of nearby points using the humidity readings. If a data value does not satisfy the temperature-humidity relationship, it will be removed from the support set. Noise that often exists in sensor measurements during the gathering process also contributes to the relative change in the estimated temperature reading.

This experiment shows that mapping from related multiple dimensions can increase mapping accuracy and reduce the performance requirements of a single sensor.

#### 6.3.3. Experiment 3: Adaptations to Changes in the Domain Model

The aim of this experiment is the study the effectiveness of the proposed VCA in modifying the domain model to better fit the current state of the application domain. It also aims to study the effectiveness of VCA in differentiating between faulty sensor behaviour and actual changes in the domain model.

After applying heat on the brass sheet for 30 s, the obstacle length was increased. Then, we observed how the AMA used the VCA to adapt its behaviour to the emerging change in the application domain. Particularly, the bill that contains the best (closest to the actual obstacle length) detected obstacle length value, the agreed bills, and the federal laws that were examined. It was assumed here that the obstacle is continuous and the existence of this obstacle between two directly communicating nodes will break the wireless links between them. In addition, 30% of the nodes were configured to act as faulty nodes in each experimental run and *υ* was assigned different values. Faulty nodes were allowed to propose bills that carry incorrect changes in the application domain. Finally, it was studied how the choice of *υ* will affect the VCA ability to discover bills generated by faulty nodes.

Due to the effect that network density and nodes distribution have on the mapping accuracy results, five simulation runs are combined to estimate uncertainties in the simulations. In each simulation run, sensor nodes were randomly positioned in the simulation window. At each point, the measured metric of the five runs was averaged.

**Figure 19:**
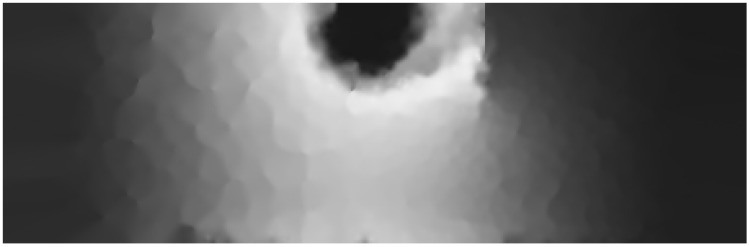
Heat map generated by AMA with 2 *cm* obstacle length.

Results in Table 1 show that VCA has always detected the obstacle length accurately, but the best proposed bill was not always agreed within the cluster. This is partially due to the properties of the used clustering protocol, which is able to deal with obstacles (see [19]). Nonetheless, the average VCA agreed bills in the three mapping runs were 53.91 pixels, which is close to the actual obstacle length (60 pixels). Adapting the mapping service to the new obstacle length improves the produced map quality. Figure 19 shows the heat map generated by AMA using the initial obstacle length. Visually, this map does not totally reflect the current state of the application domain compared to Figure 15 (map with obstacle length equal to 60 pixels). Quantitatively, the RMSD difference from Figure 15 was increased by 1.0.

**Table 1:** The obstacle length (in pixels) in the best proposed bill β and the agreed bill Å in 3 AMA runs at *D* nodes density. *N* the number of proposed bills in each run.

	Run1			Run 2			Run 3		
*D*	*N*	β	Å	*N*	β	Å	*N*	β	Å
100	16	43.46	43.46	14	53.26	53.26	13	48.64	34.07
500	15	59.33	53.96	16	59.82	58.65	11	60.0	53.42
1000	17	59.57	52.0	19	60.0	57.74	14	60.0	52.0

In the three mapping runs, zero bills became state laws. This is because all the changes in the application domain were local to part of the network and the majority of the clusters did not sense these changes. This illustrates the benefit of localising the VCA. Table 1 shows the results of VCA testing with 100 and 500 network densities each with results from three different random topologies.

Table Table 2 shows the number of bills proposed by faulty nodes and how many of those bills were agreed. First, the number of bills generated by the faulty sensors in each experimental run with different values for *υ* were counted. The values tested were υ=40%, υ=50%, and υ=60%. It was found that the υ=50% gave the minimum number of agreed faulty bills while avoiding the false positives we get with the υ=40%. Whereas the υ=60% caused many correct bills to be rejected. The results shown in Table 2 were generated using υ=50%.

**Table 2:** The number of bills F proposed by faulty nodes and the number of times they were successfully agreed Å.

	Run 1	Run 2	Run 3
F	2	2	1
Å	0	1	0

This experiment shows that VCA helps to adapt to some changes in the domain model in a distributed manner. It also shows that the optimal value for *υ* is 50%. This experiment is an instance of the general case studied in Experiment 2 , particularly, the nearest neighbour triangulation RF connectivity map is used as one dimension to predict the heat map.

Table 3 summarises all the evaluation experiments and the key obtained results.

**Table 3:** Summary of evaluation experiments and key results.

Experiment	Aim	Key Findings
Centralised mapping service: Experimental isopleth generation	Highlight the potential use of the mapping service	Map reconstruction is achieved using less than 1% of the original data
Distributed mapping service: Energy consumption	Study its efficiency in terms of energy consumption	It reduces the mapping traffic by up to 70% and 30% compared to the centralised and the suppression-based mapping respectively
Distributed mapping service: Quality of the reconstructed maps	Study its efficiency in terms of the quality of the produced map	The difference between the quality of produced maps by the distributed and centralised mapping algorithms is just 1.2%
AMA: Incorporation of the domain model in mapping	Study the effect of integrating the domain model knowledge into the mapping service	The obtained map conserves perfectly the global appearance and many of the details of the original map with 98.5% less data
AMA: Mapping from related multiple dimensions	Study how it can improve mapping performance by overcoming some of the limitations of generating a map from a single sensory modality	Increased mapping accuracy and reduced the performance requirements of a single sensor
AMA: Adaptations to changes in the domain model	Study the effectiveness of VCA in: (1) modifying the domain model; (2) differentiating between faultysensor behaviour and actualchanges in the domain model.	VCA helps to adapt to some changes in the domain model in a distributed manner. *v* optimal value is 50%

## 7. Conclusions

In this work, we have taken an *evolutionary* approach to address the issues of efficient sense data extraction, processing and visualisation using the capabilities of a standard WSN. Rather than focusing only on a single component of the WSN architecture to improve efficiency, a cross-component approach that maximises the utilisation of data offered by various WSN components has been presented. Initially, a distributed mapping service was developed to build and maintain maps in a distributed manner. This service was extended and generalised to exploit the fact that information about a particular event of interest is usually captured in multiple sensed modalities. The new mapping framework, called AMA, also utilises the application domain for mapping independent, related multivariate data. The utilisation of the application model proved to leverage computational power to simulate, visualise, manipulate, predict and gain intuition about the phenomenon being studied. To enable accurate mapping in dynamic environments that impose varying functional and performance requirements, a self-adaptation extension, called VCA, has been added to automatically adapt the mapping service behaviour to environmental changes. Finally, the distributed VCA made AMA more resilient to faults by helping to differentiate between environmental changes and faulty node behaviour. A major contribution here is the development of a theoretical and practical understanding of WSN system development and adaptivity with respect to the specific context in which the system is deployed. A firm foundation has been presented upon which to build new WSN services and applications.

In the future, we plan to design and implement a dynamic communication scheme between the mapping framework and the middleware. This allows achieving the desired quality of service of the whole system when there is more than one application running simultaneously. Another route we intend to follow is to identify suitable methods to covert semantic information to syntactic information to achieve better description and attribution of environmental conditions and events, validation and extension of the application specific error models, and the development of a data association technique to handle the uncertainty of the measurement origin. One challenges that needs attention is to guarantee temporal and spatial correlation among data sources while the data is integrated and distributed at the same time; the current framework assumes spatio-temporal data correlation among sensory data. An interesting avenue to follow is to test the presented mapping service with existing energy efficient routing protocols from the literature to study their impact on the mapping service total energy consumption. Finally, there is a great potential for the mapping service in surveillance systems, flood management, and other application areas; we intend implement these applications on top of the presented mapping approach to verify its efficiency in numerous context.
